# Ethics of research on stem cells and regenerative medicine: ethical guidelines in the Islamic Republic of Iran

**DOI:** 10.1186/s13287-020-01916-z

**Published:** 2020-09-14

**Authors:** Leila Afshar, Hamid-Reza Aghayan, Jila Sadighi, Babak Arjmand, Seyed-Mahmoud Hashemi, Mohsen Basiri, Reza Omani Samani, Mohammad Kazemi Ashtiani, Seyed-Ali Azin, Ensiyeh Hajizadeh-Saffar, Ehsan Shamsi Gooshki, Amir-Ali Hamidieh, Mohammad-Reza Rezania Moallem, Seyed-Mohammad Azin, Sadegh Shariatinasab, Mehdi Soleymani-Goloujeh, Hossein Baharvand

**Affiliations:** 1 Department of Medical Ethics, Shahid Beheshti University of Medical Sciences, Tehran, Iran; 2 Cell therapy and Regenerative Medicine Research Center, Endocrinology and Metabolism Molecular Cellular Sciences Institute, Tehran University of Medical Sciences, Tehran, Iran; 3 Department of Health Promotion, Health Metrics Research Center, Institute for Health Sciences Research, ACECR, Tehran, Iran; 4 Department of Immunology, School of Medicine, Shahid Beheshti University of Medical Sciences, Tehran, Iran; 5 Department of Stem Cells and Developmental Biology, Cell Science Research Center, Royan Institute for Stem Cell Biology and Technology, ACECR, Banihashem Sq., Banihashem St., Ressalat highway,1665659911, P.O. Box: 16635-148, Tehran, Iran; 6 Department of Medical Ethics and Law, Reproductive Biomedicine Research Center, Royan Institute for Reproductive Biomedicine, ACECR, Tehran, Iran; 7 Department of Cell Engineering, Cell Science Research Center, Royan Institute for Stem Cell Biology and Technology, ACECR, Tehran, Iran; 8 Reproductive Biotechnology Research Center, Avicenna Research Institute, ACECR, Tehran, Iran; 9 Department of Regenerative Medicine, Cell Science Research Center, Royan Institute for Stem Cell Biology and Technology, ACECR, Tehran, Iran; 10 Medical Ethics and History of Medicine Research Center, Department of Medical Ethics, Faculty of Medicine, Tehran University of Medical Sciences, Tehran, Iran; 11 Pediatric cell therapy research center, Tehran University of Medical Sciences, Tehran, Iran; 12 Department of Developmental Biology, University of Science and Culture, Tehran, Iran

**Keywords:** Clinical trial, Ethics, Guideline, Regenerative medicine, Stem cells

## Abstract

**Background:**

Regenerative medicine plays a major role in biomedicine, and given the ever-expanding boundaries of this knowledge, numerous ethical considerations have been raised.

**Main text:**

Rapid advancement of regenerative medicine science and technology in Iran, emerged the Iranian National Committee for Ethics in Biomedical Research to develop a comprehensive national ethical guideline. Therefore, the present ethical guideline which comprises eleven chapters was developed in 2019 and approved in early 2020. The titles of these chapters were selected based on the ethical considerations of various aspects of the field of regenerative medicine: (1) ethical principles of research on stem cells and regenerative medicine; (2) ethical considerations for research on stem cells (embryonic stem cells, epiblast stem cells, tissue-specific stem cells, stem cells derived from transdifferentiation, induced pluripotent stem cells [iPSCs], germline pluripotent stem cells, germline stem cells, and somatic cell nuclear transfer [SCNT] stem cells); (3) ethical considerations for research on somatic cells in regenerative medicine (adult somatic cells, fetal tissue somatic cells, and somatic cells derived from pregnancy products [other than fetus]); (4) ethical considerations for research on gametes in regenerative medicine; (5) ethical considerations for research related to genetic manipulation (human and animal) in regenerative medicine; (6) ethical considerations for research on tissue engineering in regenerative medicine; (7) ethical considerations for pre-clinical studies in regenerative medicine; (8) ethical considerations for clinical trials in regenerative medicine; (9) ethical considerations for stem cells and regenerative medicine bio-banks; (10) ethical considerations for privacy and confidentiality; and (11) ethical considerations for obtaining informed consent.

**Conclusion:**

This article discusses the process of developing the present ethical guidelines and its practical points. We hope that it can play an important worldwide role in advancing ethics of research on stem cells and regenerative medicine.

## Introduction

Regenerative medicine, especially the stem cells, plays a major role in biomedicine and introduces tremendous capacity for replacement, engineering, repair, or regeneration of cells, tissues, or organs to restore or maintain their normal functions [1, 2]. The rapid expansion of regenerative medicine science and its product commercialization has created numerous ethical concerns and considerations [3, 4]. The development and implementation of relevant research ethical guidelines has received special attention in many countries in an attempt to address these concerns, in addition to developing guidelines and standards for the production and use of stem cells and regenerative medicine products.

The first national ethical guideline on stem cell research in Iran was issued in 2013. Advances in regenerative medicine and the number of related clinical trials indicated a serious need to update this ethical guideline. In this regard, Iranian National Committee for Ethics in Biomedical Research was commissioned to develop an updated comprehensive guideline for regenerative medicine. The updated version of ethical guideline was prepared in 2019 and formally approved by the committee in 2020. It was attempted to cover all areas of research that pertained to the numerous aspects of regenerative medicine. However, due to the prominent role of stem cells in regenerative medicine, the term “stem cells” is mentioned separately in title of the present ethical guidelines.

## Main text

The present ethical guideline has been developed by a research team and designed as a qualitative study. Research group specialists included PhD in cell and developmental biology, medical ethics, physicians, immunology, molecular genetics, polymer engineering, social medicine, medical biotechnology, and law. Data were collected through group discussions and expert panels. The latest version was presented to the Iranian National Ethics Committee for Ethics in Biomedical Research for final review and approval. Supplementary Table 1 provides a list of all ethical codes of the present guideline, which is comprised of eleven chapters. A number of the important points in each chapter are presented as follows:

1. The first chapter pertains to general principles and is based on the ethical principles of biomedical research [5], which focuses on the challenges of stem cells and regenerative medicine. The principles consist of “integrity and validity of research activities,” “transparency,” “social justice,” “primacy of the participant’s health,” “risk/benefit assessment,” “optimal use of biological samples,” “respecting the rights of all participants in the research process,” “ethical principles in research with laboratory animals,” and “prohibition of commercial relations in stem cell research.”
2. The second chapter contains the ethical considerations of research on stem cells as well as research on products derived from stem cells.
  2.1 Pluripotent stem cells, like embryonic stem cells, have important ethical considerations and challenges [6]. In this guideline, some of the ethical considerations for embryonic stem cell use are as follows: (a) One of the authorized resources to produce embryonic stem cells is a human embryo less than 14 days of age after in vitro fertilization (IVF), which was legally obtained from surplus or non-transferable IVF embryos from infertility treatment or pre-implantation genetic diagnosis. (b) Transplantation of embryonic stem cells from a human to a “human embryo or fetus” is prohibited. (c) Transplantation of embryonic stem cells (and other pluripotent stem cells) from an animal to a “human” or to a “human embryo or fetus” and chimera formation are not allowed, given the ethical considerations that surround transplantation of pluripotent cells of various animal species.
  2.2 Research on multipotent stem cells, such as tissue-specific stem cells, has less ethical considerations than pluripotent stem cells. In this guideline, tissue-specific stem cells are classified as adult stem cells (tissues after birth), fetal stem cells, and stem cells derived from pregnancy products (e.g., amniotic membranes, umbilical cord, placenta, and amniotic fluid). Although the fetus is considered a pregnancy product, its ethical considerations are mentioned separately due to the importance of these considerations that pertain to the fetus. Based on this guideline, only a human fetus obtained from a therapeutic abortion, spontaneous abortion, or stillbirth can be used as a source to produce fetal tissue stem cells. The individuals or groups who decide the need for abortion should be independent from the research team and have no other conflict of interests.
  2.3 In this guideline, the ethical considerations of “stem cells derived from transdifferentiation” are mainly similar to the ethical considerations of “tissue-specific stem cells.”
  2.4 Induced pluripotent stem cells (iPSCs) are functionally similar to embryonic stem cells. The ethical considerations of these cells are less than embryonic stem cells only in the source of cell production. In this guideline, authorized resources for production of iPSCs are similar to those allowed for “tissue-specific stem cells.” However, ethical considerations on research application of iPSCs are similar to research application of “embryonic stem cells.”
  2.5 Germline stem cells and germline pluripotent stem cells are a type of cell-like pluripotent stem cell; however, their differentiation capacity is less than both the embryonic stem cells and iPSCs. The ethical considerations on research application of these cells are similar to research application of “embryonic stem cells.”
  2.6 Somatic cell nuclear transfer (SCNT) stem cells are produced by transferring the nucleus of a somatic cell into an enucleated egg (oocyte) and behave like embryonic stem cells. There are many ethical considerations for SCNT research [7, 8]. In this guideline, important ethical considerations include the following: (a) Production of SCNT stem cells with the aim of “human therapeutic cloning” is allowed, but the resulting embryo can survive only until the age of 14 days and then must be discarded. (b) The production of SCNT stem cells with the aim of “human reproductive cloning” is prohibited. (c) Human somatic cell nuclear transfer into an “animal egg” and production of a cytoplasmic hybrid (cybrid) are allowed. However, the resulting embryo is only allowed to survive until the age of 14 days, and then, it must be discarded. (d) Animal somatic cell nuclear transfer into a “human egg” and production of a cybrid is prohibited.
3. The third chapter pertains to ethical considerations for research on somatic cells in regenerative medicine. These considerations are somewhat similar to the ethical considerations of tissue-specific stem cells.
4. The fourth chapter presents ethical considerations for research on gametes in regenerative medicine. According to this guideline, important authorized resources to supply human gametes for research include excess eggs from infertility treatments (egg sharing and unusable eggs) and eggs obtained from in vitro transformation of stem cells. Furthermore, fertilization of a human gamete with an animal gamete and production of a hybrid is prohibited.
5. Chapter five addresses important ethical considerations for research related to genetic manipulation in regenerative medicine [9, 10]. Some considerations include the following: (a) Researchers are only allowed to genetically manipulate human surplus or non-transferable IVF embryos obtained from infertility treatments or pre-implantation genetic diagnosis. This embryo is not allowed to be transferred to the uterus, and this manipulated human embryo is only allowed to survive for 14 days after IVF and must be discarded after this period. (b) In vivo genetic manipulation of a human fetus is prohibited. (c) Clinical trials of genetic manipulation in children (less than 18 years of age) are prohibited. This type of trial is only allowed in children diagnosed with life-threatening diseases that have no alternative treatments. (d) Genetic manipulation for the purpose of human enhancement and eugenics and to produce a transgenic human is prohibited. (e) Genetic manipulation that causes suffering of animals or disruptions to their normal life process by changing their traits and characteristics should be avoided.
6. Chapter six deals with the ethical considerations for research on tissue engineering [11]. Clinical trials of tissue-engineered products should be performed by taking into consideration the degree of interaction with the body, frequency, and duration of application as well as transplantation conditions. Any hazardous material that has been used in the production of biomaterials should be disposed of or recycled with consideration for all safety standards.
7. Chapter seven includes the ethical considerations of pre-clinical studies in regenerative medicine. This chapter focuses on animal ethics and is classified under the following headings: choice of a suitable animal model, interventions in animal, animal housing and place of research, veterinary personnel and care, and the procedure to end working with the animals. Some considerations include the following: (a) Animal species can be used if they are bred to be used in research studies. (b) Primate animals can be used only in studies carried out with an approach to the prevention, diagnosis or treatment of a disability or clinical conditions that are potentially hazardous to human life, and when there is sufficient scientific justification for the study and research purpose, which could not be achieved by using other animal species. (c) Surgery or dissection of animals or other interventions that inflict similar surgical pain on live non-anesthetized animals or those that do not undergo full analgesia is strictly prohibited.
8. Chapter eight is the ethical guideline for clinical trials on stem cells and regenerative medicine. In addition to the potential benefits of the use of a variety of cells and cell-based products in clinical trials, there are concerns about possible complications of their application, ethical challenges about the design and conduct of the clinical trials, and preparation of cells from a variety of sources such as human embryos [12, 13]. According to this guideline, stem cells’ and regenerative medicine products’ clinical trials can be only carried out when there is no effective treatment for the disease at the time of the research or it is expected that the intervention will produce better results and fewer complications compared to the existing therapies, and based on the pre-clinical results and available scientific evidence. In cases where there is an effective treatment, risks of interventions related to stem cells and regenerative medicine must be at a minimum and justifiable level, and its use should lead to potential advantages such as fewer complications, shorter recovery time, increased quality of life, and reduced costs over an extended period of time. This guideline also emphasizes that the results of animal studies are not necessarily indicative of cell behavior in humans. Therefore, safety and efficacy studies in animals may not correctly predict responses that occur in humans. For this reason, it is recommended that all necessary measures be taken to precisely examine and confront possible adverse effects in humans. The transplanted cells are living organisms and may remain in the body for an extended period of time; therefore, accurate continuous and precise monitoring of subjects is required after transplantation.
9. Chapter nine addresses the ethical guideline for stem cells and regenerative medicine bio-banks [14]. Biological samples must be kept safely and should be labeled or coded. The samples should be preserved according to standard manners to ensure that they are not damaged or lost, and only authorized persons have access to bio-banks. Informed consent must be obtained before the biological samples are obtained from the donor or his/her legal representative. The bio-bank staff that has access to samples and biological data must respect privacy policies and avoid data transfer without legal authorization.
10. Chapter ten deals with the ethical guideline for privacy and confidentiality of information. Protection of participants’ privacy in research on stem cells and regenerative medicine as well as confidentiality of their information is taken into account.
11. Chapter eleven is the ethical guideline for obtaining informed consent. Ethical considerations have been formulated in terms of “informed consent to donate biological samples” and “informed consent to participate in research.”

## Conclusion

The present ethical guidelines address internationally related ethical considerations according to national experts’ opinions and will be revised and updated over time, given the rapid increase in development of knowledge of stem cells and regenerative medicine. We hope that it can play an important worldwide role in advancing ethics of research on stem cells and regenerative medicine.

## Supplementary information

**Additional file 1: Supplementery Table 1.** Ethical guideline for research on stem cells and regenerative medicine in the Islamic Republic of Iran.

## Data Availability

Not applicable.

## Abbreviations

IVF: In vitro fertilization
iPSCs: Induced pluripotent stem cells
SCNT: Somatic cell nuclear transfer
ACECR: Iranian Academic Center for Education, Culture, and Research
