# Supplementary material for: Ethics of research on stem cells and regenerative medicine: ethical guidelines in the Islamic Republic of Iran
Source: Stem Cell Res Ther. 2020 Sep 14;11:396. doi: 10.1186/s13287-020-01916-z (PMC7489032; doi:10.1186/s13287-020-01916-z)
Supplement: Supplementary file 1 — Additional file 1: Supplementery Table 1. Ethical guideline for research on stem cells and regenerative medicine in the Islamic Republic of Iran. [file 13287_2020_1916_MOESM1_ESM.docx]

**Supplementery Table 1.**  Ethical guideline for research on stem cells and regenerative medicine in the Islamic Republic of Iran.

| **Chapter** | **Description** | |
| --- | --- | --- |
| **Chapter 1** | Ethical principles of research on stem cells and regenerative medicine | |
| **1.1** | Integrity and validity of research activities | |
| 1.1.1 | The primary objective purpose of researches on stem cells and regenerative medicine is to achieve scientific understanding and produce credible evidence to meet the health and medical needs of the society. | |
| 1.1.2 | These researches must be designed and implemented by experienced researchers so that society can trust their results. These results should be reliable, accessible, and responsive to the health and medical needs of the society. Key processes to ensure such integrity include independent peer reviews, reproducibility, and transparency in all steps of the research. | |
| 1.1.3 | Researchers must be familiar with national codes of ethics, laws, and regulations in their research field and their possible changes, and act in accordance with these regulations during all stages of research. | |
| 1.1.4 | Each research proposal must be reviewed and approved by a qualified scientific committee and then by a research ethics committee. It is possible to start the research process only after obtaining the research ethics certificate, issued by the research ethics committe. | |
| 1.1.5 | All clinical trials in the field of regenerative medicine must have been registered in the Iranian Registry of Clinical Trials. | |
| **1.2** | Transparency | |
| 1.2.1 | Researchers who work in the field of stem cells and regenerative medicine should place accurate scientific information to the interested parties such as other researchers, patients, clinicians, research sponsoring organizations, and health system policy makers in a timely manner. Reporting any uncertainty about the safety and effectiveness of the investigational procedures is also subject to this principle. | |
| 1.2.2 | Researchers and their sponsors are required to provide conditions and opportunities to share ideas, methods, materials, and information. | |
| **1.3** | Social justice | |
| 1.3.1 | Benefits of stem cells and regenerative medicine research should be reasonably accessible and address the community health needs. | |
| 1.3.2 | Variables (age, gender, and ethnicity) should be considered when selecting participants for research studies. | |
| 1.3.3 | Research that has no potential outcomes for individuals or communities under study should not be performed on those individuals or communities. | |
| 1.3.4 | The cost of guaranteeing the safety and effectiveness of interventions and offsetting adverse events that are attributable to research on stem cells and regenerative medicine should not be paid by participants and their health insurance organizations. The researcher, company, sponsor, and research insurance organizations should compensate these costs. According to the Guideline of the National Biomedical Research Ethics Committee, entitled "Patient Funded Clinical Trials" only in rare situations charging of clinical trial’s participants is permitted. | |
| **1.4** | Primacy of the participant’s health | |
| 1.4.1 | Researchers are responsible for each participant’s health and welfare. Vulnerability of human beings should be considered while using and advancing stem cells and regenerative medicine knowledge and its related technologies. Vulnerable people and groups should be protected. | |
| 1.4.2 | The probability of the research benefits in the future and its process should not affect the health status of research participants. While defining the concept of vulnerability in stem cell and regenerative medicine research, it is necessary to pay attention to the following points:   - What individual(s) are potentially at risk of injury, damage, and error? - What are the forms and types of vulnerability? - Which underlying factors require special attention? - Has the risk of participating in research compared with its potential benefits been properly assessed? - What additional protective actions are required for participants, and who is responsible for these protective measures during high-risk conditions? | |
| **1.5** | Risk/benefit assessments | |
| 1.5.1 | In all steps of research on stem cells and regenerative medicine, the risk-benefit of participating in the research must be assessed. The research should be allowed only if the potential benefits of participating outweigh any potential risks. This also includes cell, tissue, or organ donors. | |
| 1.5.2 | Although there may not be many physical complications from the donation, researchers should consider the possibility of other injuries, including economic, psychological or social risks such as the risks of possible disclosure of research results and other information when assessing the risk/benefit ratio. Therefore, "minimal acceptable risk" should be defined in each research protocol. | |
| 1.5.3 | Compliance with safety and laboratory regulatory documents approved by the Iran Food and Drug Administration at all stages of procurement, collection, processing, preservation, and transplantation of cells and their derivatives is mandatory. | |
| **1.6** | Optimal use of biological samples | |
| 1.6.1 | Researchers should treat human and animal body parts with respect. | |
| 1.6.2 | Human biological samples including cells, organs, and tissues should be "donated"; and any type of research on these samples must be carried out with respect and transparency. Researchers should note that a participant’s personal, cultural, and religious differences can lead to different interpretations of the body or its parts, and these different interpretations must be taken into consideration. | |
| 1.6.3 | The researcher has the responsibility to approach the individual in a respectful and trustable manner to the donation as an altruistic act. This approach is possible by providing a high level of transparency in all stages of research - from the process of obtaining the informed consent to the possibility of future use of samples and publication of research findings. | |
| 1.6.4 | To enable the best usage of samples, proper planning should be undertaken to ensure appropriate use of new tissues or full usage of the existing tissues. If the previously collected samples can be used, then the researcher should abstain from obtaining new samples. This could be possible by providing full and adequate information from the available resources. Selecting proper storage and preparation procedures, better sampling, and obtaining valid informed consent of the donors will ensure proper use of the samples. | |
| 1.6.5 | The samples should not be stored for an extended period of time without any purpose or destroyed without justification. | |
| **1.7** | Respecting the rights of all participants in the research process and maintaining their moral standings | |
| 1.7.1 | It is necessary to respect the rights of all subjects in stem cell and regenerative medicine research. The most important are research participants and donors of biological samples. Obtaining an informed consent is an example of this respect. | |
| 1.7.2 | The principle of respect should be interpreted such that the rights of all individuals, including cell and tissue donors, participants, and researchers is taken into consideration. | |
| 1.7.3 | Researchers should respect the confidentiality of the medical and personal information of research participants. | |
| 1.7.4 | Research on humans, particularly human embryo and fetus must be carried out in compliance with ethical and juridical guidlines. | |
| 1.7.5 | Human research should not lead to genetic mutations, be transferable to the future generations, or cause changes in human identity. | |
| 1.7.6 | Human research should not result in the creation of new species from the combination of humans and other animals. | |
| **1.8** | Ethical principles in research that uses laboratory animals | |
| *All researches conducted on laboratory animals should be designed with regards to four principles: ethical behavior, replacement, reduction, and refinement*. | | |
| 1.8.1 | | Principle of ethical behavior: Most animals experience pain, suffering, despair, depression, anxiety, and panic during a study. Therefore, all individuals involved in the development, maintenance, and use of laboratory animals have a responsibility to treat them ethically. These individuals must ensure the welfare of laboratory animals and perform their tasks based on professional ethics and scientific principles. |
| 1.8.2 | | Principle of replacement: If there is an alternative solution other than the use of animals in research, then it is not ethically permissible to use animals. In these cases, the use of alternative methods takes precedence over the use of living animals. For example, living organisms of a lower evolutionary category or non-animals such as plants, microorganisms, protozoa, parasites, computerized and simulation methods, mathematical and statistical calculations, in vitro tests, and other valid scientific methods can be used. |
| 1.8.3 | | Principle of reduction: The minimum number of living animals should be used in all research interventions. However, this reduction should not lead to an increase in suffering by these animals, as well as repeating the tests and the use of more laboratory animals, or damage to other living creatures by producing unreliable scientific data. |
| 1.8.4 | | Principle of refinement: Researchers should try to provide better conditions in animal houses and enhance their welfare. Laboratory animals should be treated such that the animals have minimal suffering. Appropriate intervention methods, knowing the theoretical and practical principles of working with laboratory animals, and correct selection of the animal species can enhance the animal's welfare. |
| **1.9** | | Prohibition of commercial relations in stem cell research |
| 1.9.1 | | The human body and any of its parts should not be subject to commercial activities or financial benefits. The researcher should not sell human biological samples and the participants should not donate their biological samples because of financial incentive. However, it is accepted to compensate for a participant’s usual costs such as travel and time, and the cost of preparation and preservation of biological samples. |
| **Chapter 2** | | Ethical guideline for research on stem cells |
| **2.1** | | Embryonic stem cells |
| 2.1.1 | | **Authorized resources to produce and supply embryonic stem cells** |
| 2.1.1.1 | | Human embryos up to 14 days after in vitro fertilization that have been derived legally from surplus or non-transferable IVF embryos due to infertility treatment or pre-implantation genetic diagnosis.  *Note*: The duration for embryo freezing is not included in the age calculation for human embryos. |
| 2.1.1.2 | | Human Parthenon embryos up to 14 days (the result of parthenogenesis) produced with the aim of therapy. |
| 2.1.1.3 | | Embryos of laboratory animals (in compliance with the Guideline for the Care and Use of Laboratory Animals, approved by the Iranian Ministry of Health and Medical Education). |
| 2.1.1.4 | | Embryonic stem cell lines from bio-banks (in compliance with ethical considerations listed in the chapter: Ethical guideline for stem cells and regenerative medicine bio-banks). |
| 2.1.2 | | **Ethical considerations on the production and supply of embryonic stem cells** |
| 2.1.2.1 | | The human embryo produced by in vitro fertilization for "research purposes" is prohibited. |
| 2.1.2.2 | | Extraction of embryonic stem cells from human embryos derived from in vivo fertilization is prohibited. |
| 2.1.2.3 | | Extraction of embryonic stem cells from human embryos older than 14 days that have been derived from in vitro fertilization is prohibited. |
| 2.1.2.4 | | Researchers who use embryonic stem cells for research purposes should have no conflict of interest, and should not be members of an infertility treatment team and producers of in vitro fertilization-derived embryos. |
| 2.1.2.5 | | Human embryos used to produce embryonic stem cells should not be obtained through trading and any intention for material benefits. |
| 2.1.2.6 | | Researchers are authorized to kill animals to extract embryonic stem cells from animal embryos only when they follow the Guideline for the Care and Use of Laboratory Animals (approved by Iranian Ministry of Health and Medical Education). It is prohibited to kill pregnant animals to extract embryonic stem cells from their embryos in situations where there is a possibility that a cesarean can be performed. |
| 2.1.3 | | **Ethical considerations on research application of embryonic stem cells** |
| 2.1.3.1 | | In vivo transplantation of "undifferentiated" embryonic stem cells from one human to another human is prohibited. |
| 2.1.3.2 | | Transplantation of embryonic stem cells from a human to a "human embryo or fetus" is prohibited. |
| 2.1.3.3 | | Transplantation of embryonic stem cells from an animal to a "human" or to a "human embryo or fetus", and chimera formation are prohibited. |
| 2.1.3.4 | | Transplantation of embryonic stem cells from a human to an "animal embryo" and chimera formation is allowed; however, this chimera can be permitted to survive until 14 days after the transplant and must be discarded after this period. |
| 2.1.3.5 | | Fertilization of gametes derived from embryonic stem cells of animals with "human gametes" and the production of a hybrid is prohibited. |
| 2.1.3.6 | | It is allowed to perform in vitro fertilization of gametes derived from human embryonic stem cells with "gametes of animals" to evaluate the fertilization ability of these cells. However, after fertilization, this hybrid must be discarded. |
| **2.2** | | Epiblast stem cells |
| 2.2.1 | | The authorized resources to produce epiblast stem cells and its ethical considerations on the production and research applications are similar to "embryonic stem cells". |
| **2.3** | | Tissue-specific stem cells |
| **2.3.1** | | Adult stem cells (tissue after birth) |
| 2.3.1.1 | | **Authorized resources to produce and supply adult stem cells** |
| 2.3.1.1.1 | | Living tissue donors. |
| 2.3.1.1.2 | | Human tissue remains resulting from surgery, diagnostic, and other treatment methods. |
| 2.3.1.1.3 | | Tissue from brain-dead donor. |
| 2.3.1.1.4 | | Cadaveric tissue donors.  *Note:* It is possible to use any type of human tissue as long as the Ethical Guideline for Research on Human Organ and Tissue (approved by the Iranian Ministry of Health and Medical Education) is observed. |
| 2.3.1.1.5 | | Tissue of laboratory animals (in compliance with the Guideline for the Care and Use of Laboratory Animals, approved by the Iranian Ministry of Health and Medical Education). |
| 2.3.1.1.6 | | Adult stem cell lines from bio-banks (in compliance with ethical considerations listed in the chapter: Ethical guideline for stem cells and regenerative medicine bio-banks). |
| 2.3.1.2 | | **Ethical considerations on the production and supply of adult stem cells** |
| 2.3.1.2.1 | | Production of adult stem cells from the tissue of a living donor is allowed only if there is "minimal acceptable risk" and there is a likelihood for compensation of possible damage to the donor's organs and lack of causing damage to his/her physiological functions. |
| 2.3.1.2.2 | | The donor must be followed in terms of any incidences of complications that result from donation process and receive free medical servicess if necessary. Follow-up duration should be determined. |
| 2.3.1.2.3 | | Human adult stem cells used in research should not have been obtained through trading and any intention for material benefits. |
| 2.3.1.3 | | **Ethical considerations on research application of adult stem cells** |
| 2.3.1.3.1 | | Transplantation of adult stem cells from a human to a ''human embryo" is prohibited. |
| 2.3.1.3.2 | | Transplantation of adult stem cells from an animal to a "human embryo or fetus" and chimera formation is prohibited. |
| 2.3.1.3.3 | | Fertilization of gametes derived from adult stem cells of animals with "gametes of humans" and the production of a hybrid is prohibited. |
| 2.3.1.3.4 | | It is allowed to perform in vitro fertilization of gametes derived from human adult stem cells with "gametes of animals" to evaluate the fertilization ability of these cells. This hybrid must be discarded after fertilization. |
| **2.3.2** | | Fetal tissue stem cells |
| 2.3.2.1 | | **Authorized resources to produce and supply fetal tissue stem cells** |
| 2.3.2.1.1 | | Human fetuses obtained from therapeutic abortions, spontaneous abortions, or stillbirths. |
| 2.3.2.1.2 | | Fetuses of laboratory animals (in compliance with the Guideline for the Care and Use of Laboratory Animals, approved by the Iranian Ministry of Health and Medical Education). |
| 2.3.2.1.3 | | Fetal tissue stem cell lines from bio-banks (in compliance with ethical considerations listed in the chapter: Ethical guideline for stem cells and regenerative medicine bio-banks). |
| 2.3.2.2 | | **Ethical considerations on the production and supply of fetal tissue stem cells** |
| 2.3.2.2.1 | | Individuals or groups that decide the need for a human abortion should be independent from the research team and have no conflict of interest. |
| 2.3.2.2.2 | | Use of a human fetus to obtain stem cells should have no impact on maternal decision-making to undergo an abortion. |
| 2.3.2.2.3 | | The aborted fetus should be treated with respect during the study. The fetus should be in a proper container, all fetal components are placed together, and the least damage is imposed on its body. In terms of obtaining consent from the parents and at their request, if possible, the fetus should be returned to them after the end of the study or be buried by the research group with respect to legal formalities and religious beliefs. |
| 2.3.2.2.4 | | The method of research on human and animal fetal tissue should be pre-determined in terms of "type and amount of tissue needed and the time during which the fetus would be retained". The Research Ethics Committee should be informed about these information through project application forms. |
| 2.3.2.2.5 | | A human fetus used for research should not have been obtained through trading and any intention for material benefits. |
| 2.3.2.2.6 | | Researchers are only authorized to kill animals to extract fetal stem cells according to the Guideline for the Care and Use of Laboratory Animals (approved by the Iranian Ministry of Health and Medical Education). It is prohibited to kill pregnant animals to extract fetal stem cells from their fetuses in situations where the possibility for a cesarean exists. |
| 2.3.2.3 | | **Ethical considerations on research application of fetal tissue stem cells** |
| 2.3.2.3.1 | | Ethical considerations on research application of fetal tissue stem cells are similar to the research application of "adult stem cells". |
| **2.3.3** | | Stem cells derived from pregnancy products (other than fetus) |
| 2.3.3.1 | | **Authorized resources to produce and supply stem cells derived from pregnancy products** |
| 2.3.3.1.1 | | Human pregnancy products derived from the birth of a newborn. |
| 2.3.3.1.2 | | Human pregnancy products derived from a therapeutic abortion, spontaneous abortion, or stillbirth. |
| 2.3.3.1.3 | | Pregnancy products derived from laboratory animals (in compliance with the Guideline for the Care and Use of Laboratory Animals, approved by the Iranian Ministry of Health and Medical Education). |
| 2.3.3.1.4 | | Pregnancy products stem cell lines from bio-banks (in compliance with ethical considerations listed in the chapter: Ethical guideline for stem cells and regenerative medicine bio-banks). |
| 2.3.3.2 | | **Ethical considerations on the production and supply of stem cells derived from pregnancy products** |
| 2.3.3.2.1 | | If the pregnancy products are obtained from human abortions, then the person or team that determines the need for the abortion should be independent from the research team. |
| 2.3.3.2.2 | | If the pregnancy products are obtained from human abortions, the use of pregnancy products to obtain stem cells should not have an impact on maternal decision-making to undergo an abortion. |
| 2.3.3.2.3 | | Use of human amniotic fluid during pregnancy for research purposes is only possible through simultaneous diagnostic or therapeutic procedures. |
| 2.3.3.2.4 | | Human pregnancy products used for research should not be obtained through trading and or any intention for material benefits. |
| 2.3.3.2.5 | | Killing pregnant animals to use stem cell pregnancy products is allowed only if there is no possibility of producing stem cells derived from pregnancy products from other sources and by following the Guideline for the Care and Use of Laboratory Animals (approved by the Iranian Ministry of Health and Medical Education). |
| 2.3.3.2.6 | | It is prohibited to kill pregnant animals to use stem cells derived from pregnancy products in situations where there is the possibility for a cesarean. |
| 2.3.3.3 | | **Ethical considerations on research application of stem cells derived from pregnancy products** |
| 2.3.3.3.1 | | Ethical considerations on research application of stem cells derived from pregnancy products are similar to research application of "adult stem cells". |
| **2.4** | | Stem cells derived from transdifferentiation |
| 2.4.1 | | **Authorized resources to produce and supply stem cells derived from transdifferentiation** |
| 2.4.1.1 | | Authorized resources to produce and supply tissue-specific stem cells are also applicable for stem cells derived from transdifferentiation (based on the use of adult stem cells, fetal tissue stem cells or stem cells derived from pregnancy products). |
| 2.4.1.2 | | Stem cell lines derived from transdifferentiation from bio-banks (in compliance with ethical considerations listed in the chapter: Ethical guideline for stem cells and regenerative medicine bio-banks). |
| 2.4.2 | | **Ethical considerations on the production and supply of stem cells derived from transdifferentiation** |
| 2.4.2.1 | | Ethical considerations on the production and supply of tissue-specific stem cells are also applicable for stem cells derived from transdifferentiation (based on the use of adult stem cells, fetal tissue stem cells or stem cells derived from pregnancy products). |
| 2.4.3 | | **Ethical considerations on research application of stem cells derived from transdifferentiation** |
| 2.4.3.1 | | Ethical considerations on the research application of transdifferentiated stem cells are similar to the research application of tissue-specific stem cells (based on the use of adult stem cells, fetal stem cells or stem cells derived from pregnancy products)**.** |
| 2.4.3.2 | | Transformation of human tissue cells into gametes and fertilization with their own gametes is prohibited. |
| 2.4.3.3 | | It is necessary to observe the ethical considerations listed in the chapter "Ethical guideline for research related to genetic manipulation in regenerative medicine" for the research application of stem cells derived from transdifferentiation. |
| **2.5** | | Induced pluripotent stem cells |
| 2.5.1 | | **Authorized resources to produce and supply induced pluripotent stem cells** |
| 2.5.1.1 | | Authorized resources to produce and supply tissue-specific stem cells are also applicable for induced pluripotent stem cells (based on the use of adult stem cells, fetal tissue stem cells or stem cells derived from pregnancy products). |
| 2.5.1.2 | | Induced pluripotent stem cell lines from bio-banks (in compliance with ethical considerations listed in the chapter: Ethical guideline for stem cells and regenerative medicine bio-banks). |
| 2.5.2 | | **Ethical considerations on the production and supply of induced pluripotent stem cells** |
| 2.5.2.1 | | Ethical considerations for the production and supply of tissue-specific stem cells are applicable for induced pluripotent stem cells (based on the use of adult stem cells, fetal tissue stem cells or stem cells derived from pregnancy products). |
| 2.5.3 | | **Ethical considerations on research application of induced pluripotent stem cells** |
| 2.5.3.1 | | Ethical considerations on research application of induced pluripotent stem cells are similar to research application of "embryonic stem cells**".** |
| **2.6** | | Germline pluripotent stem cells |
| 2.6.1 | | **Authorized resources to produce and supply germline pluripotent stem cells** |
| 2.6.1.1 | | Human fetuses obtained from therapeutic abortions, spontaneous abortions or stillbirths. |
| 2.6.1.2 | | Fetuses of laboratory animals (in compliance with the Guideline for the Care and Use of Laboratory Animals, approved by the Iranian Ministry of Health and Medical Education). |
| 2.6.1.3 | | The teratocarcinoma removed from cadavers or living persons after surgery. |
| 2.6.1.4 | | Germline pluripotent stem cells from bio-banks (in compliance with ethical considerations listed in the chapter: Ethical guideline for stem cells and regenerative medicine bio-banks). |
| 2.6.2 | | **Ethical considerations on the production and supply of germline pluripotent stem cells** |
| 2.6.2.1 | | If the fetal tissue is used to produce germline pluripotent stem cells, then ethical considerations on the production and supply of "fetal tissue stem cells" are applicable for the production and supply of "germline pluripotent stem cells". |
| 2.6.2.2 | | Human germline pluripotent stem cells used for research should not be obtained through trading and intention for material benefits. |
| 2.6.3 | | **Ethical considerations on research application of germline pluripotent stem cells** |
| 2.6.3.1 | | Ethical considerations on research application of germline pluripotent stem cells are similar to research application of "embryonic stem cells". |
| **2.7** | | Germline stem cells |
| 2.7.1 | | **Authorized resources to produce and supply germline stem cells** |
| 2.7.1.1 | | Remains of human testicular tissues from surgeries, diagnostic methods, and other treatment methods. |
| 2.7.1.2 | | Testicular tissues from brain-dead donors.  *Note*: It is possible to use any type of human tissue in compliance with the Ethical Guideline for Research on Organ and Tissue approved by the Iranian Ministry of Health and Medical Education. |
| 2.7.1.3 | | Testicular tissues from cadaveric donors.  *Note*: It is possible to use any type of human tissue in compliance with the Ethical Guideline for Research on Organ and Tissue approved by the Iranian Ministry of Health and Medical Education. |
| 2.7.1.4 | | Testicular tissues from laboratory animals (in compliance with the Guideline for the Care and the Use of Laboratory Animals, approved by the Iranian Ministry of Health and Medical Education). |
| 2.7.1.5 | | Germline stem cells from bio-banks (in compliance with ethical considerations listed in the chapter: Ethical guideline for stem cells and regenerative medicine bio-banks). |
| 2.7.2 | | **Ethical considerations on the production and supply of germline stem cells** |
| 2.7.2.1 | | The testicular tissue donor must undergo the follow-up procedure in terms of incidence of complications that result from donation process and receive free medical services when necessary. Duration of follow-up should be specified. |
| 2.7.2.2 | | Human germline stem cells used in research should not been obtained through trading and any intention for material benefits. |
| 2.7.3 | | **Ethical considerations on research application of germline stem cells** |
| 2.7.3.1 | | Ethical considerations on the research application of germline pluripotent stem cells are similar to research application of "embryonic stem cells". |
| **2.8** | | Somatic cell nuclear transfer (SCNT) stem cells |
| 2.8.1 | | **Authorized resources to produce SCNT stem cells** |
| 2.8.1.1 | | Human eggs (oocytes), excess need for infertility treatment (egg sharing and unusable eggs). |
| 2.8.1.2 | | Human eggs obtained from in vitro transformation of stem cells. |
| 2.8.1.3 | | Authorized resources to produce and supply tissue-specific stem cells are also applicable for stem cells derived from SCNT (based on the use of adult stem cells, fetal tissue stem cells or stem cells derived from pregnancy products). |
| 2.8.1.4 | | Stem cell lines derived from SCNT from bio-banks (in compliance with ethical considerations listed in the chapter: Ethical guideline for stem cells and regenerative medicine bio-banks). |
| 2.8.2 | | **Ethical considerations on the production and supply of SCNT stem cells** |
| 2.8.2.1 | | Production of SCNT stem cells with the intent for "human therapeutic cloning" is allowed, but the resulting embryo can only survive until the age of 14 days and then must be discarded. |
| 2.8.2.2 | | The production of SCNT stem cells with the aim of "human reproductive cloning" is prohibited. |
| 2.8.2.3 | | Ovulation induction and extraction of human eggs for research purposes is prohibited. |
| 2.8.2.4 | | Human eggs used for research should not be obtained through trading and any intention for material benefits. |
| 2.8.2.5 | | Researchers are authorized to kill animals to extract the eggs only by following the Guideline for the Care and Use of Laboratory Animals (approved by the Iranian Ministry of Health and Medical Education). It is prohibited to kill animals to extract oocytes in situations in which animal surgery is possible. |
| 2.8.2.6 | | Ethical considerations on the production and supply of tissue-specific stem cells are also applicable for SCNT stem cells (based on the use of adult stem cells, fetal tissue stem cells or stem cells derived from pregnancy products). |
| 2.8.3 | | **Ethical considerations on research application of SCNT stem cells** |
| 2.8.3.1 | | Creation of a human embryo by SCNT and placing it in the uterus and "reproductive cloning" is prohibited. |
| 2.8.3.2 | | Human somatic cell nuclear transfer into an "animal egg" and production of a cytoplasmic hybrid (cybrid) is allowed; however, the resultant embryo should only be allowed to survive until the age of 14 days and then must be discarded. |
| 2.8.3.3 | | Animal somatic cell nuclear transfer into a "human egg" and production of a cybrid is prohibited. |
| **Chapter 3** | | Ethical guideline for research on somatic cells in regenerative medicine |
| **3.1** | | Adult somatic cells (tissue after birth) |
| 3.1.1 | | **Authorized resources to produce and supply adult somatic cells** |
| 3.1.1.1 | | Adult somatic cell lines derived from bio-banks (in compliance with ethical considerations listed in the chapter: Ethical guideline for stem cells and regenerative medicine bio-banks). |
| 3.1.1.2 | | Other resources are similar to authorized resources to produce and supply "adult stem cells". |
| 3.1.2 | | **Ethical considerations on the production and supply of adult somatic cells** |
| 3.1.2.1 | | Ethical considerations on the production and supply of adult somatic cells are similar to ethical considerations on the production and supply of "adult stem cells". |
| 3.1.3 | | **Ethical considerations on research application of adult somatic cells** |
| 3.1.3.1 | | Adult somatic cells transplantation from a human to a "human embryo" is prohibited. |
| 3.1.3.2 | | Adult somatic cells transplantation from an animal to a "human embryo or fetus" is prohibited. |
| **3.2** | | Fetal tissue somatic cells |
| 3.2.1 | | **Authorized resources to produce and supply fetal tissue somatic cells** |
| 3.2.1.1 | | Fetal tissue somatic cell lines derived from bio-banks (in compliance with ethical considerations listed in the chapter: Ethical guideline for stem cells and regenerative medicine bio-banks). |
| 3.2.1.2 | | Other resources are similar to authorized resources to produce and supply "fetal tissue stem cells". |
| 3.2.2 | | **Ethical considerations on the production and supply of fetal tissue somatic cells** |
| 3.2.2.1 | | Ethical considerations on the production and supply of fetal tissue somatic cells are similar to ethical considerations on the production and supply of "fetal tissue stem cells". |
| 3.2.3 | | **Ethical considerations on research application of fetal tissue somatic cells** |
| 3.2.3.1 | | Ethical considerations on research application of fetal tissue somatic cells are similar to research application of "adult somatic cells". |
| **3.3** | | Somatic cells derived from pregnancy products (other than fetus) |
| 3.3.1 | | **Authorized resources to produce and supply somatic cells derived from pregnancy products** |
| 3.3.1.1 | | Pregnancy products somatic cell lines from bio-banks (in compliance with ethical considerations listed in the chapter: Ethical guideline for stem cells and regenerative medicine bio-banks). |
| 3.3.1.2 | | Other resources are similar to authorized resources to produce and supply "stem cells derived from pregnancy products". |
| 3.3.2 | | **Ethical considerations on the production and supply of somatic cells derived from pregnancy products** |
| 3.3.2.1 | | Ethical considerations on the production and supply of somatic cells derived from pregnancy products are similar to ethical considerations on the production and supply of "stem cells derived from pregnancy products". |
| 3.3.3 | | **Ethical considerations on research application of somatic cells derived from pregnancy products** |
| 3.3.3.1 | | Ethical considerations on research application of somatic cells derived from pregnancy products are similar to research application of "adult somatic cells". |
| **Chapter 4** | | Ethical guideline for research on gametes in regenerative medicine |
| **4.1** | | Authorized resources to produce and supply gametes |
| 4.1.1 | | Human eggs, excess need for infertility treatments (egg sharing and unusable eggs). |
| 4.1.2 | | Human eggs obtained from in vitro transformation of stem cells. |
| 4.1.3 | | Human sperm. |
| 4.1.4 | | Gametes from laboratory animals (in compliance with the Guideline for the Care and Use of Laboratory Animals, approved by the Iranian Ministry of Health and Medical Education). |
| **4.2** | | Ethical considerations on the production and supply of gametes |
| 4.2.1 | | Ovulation induction and extraction of human eggs solely for research purposes is prohibited. |
| 4.2.2 | | Human sperm for research purposes should be only obtained from married men in full compliance with religious considerations. |
| 4.2.3 | | Human gametes used for research should not be obtained through trading and any intention for material benefits. |
| 4.2.4 | | Researchers are authorized to kill animals for extraction of gametes only by following the Guideline for the Care and Use of Laboratory Animals (approved by the Iranian Ministry of Health and Medical Education). It is prohibited to kill animals in order to extract gametes when there is a possibility for animal surgery. |
| **4.3** | | Ethical considerations on research application for gametes |
| 4.3.1 | | Fertilization of human gametes with animal gametes and production of a hybrid is prohibited. |
| **Chapter 5** | | Ethical guideline for research related to genetic manipulation in regenerative medicine |
| 5.1 | | Ethical considerations on genetic manipulation of human embryo, fetus and cells |
| 5.1.1 | | Studies on genetic manipulation of embryos and gametes can help expanding knowledge on the prevention of genetic diseases. However, until the safety of the scientific methods is ensured, it is only permitted to carry out genetic manipulation on human embryo up to 14 days in surplus or non-transferable in vitro fertilized (IVF) embryos due to infertility treatments or pre-implantation genetic diagnosis. It is not allowed to transfer this embryo to the uterus and the manipulated human embryo should only be allowed to survive for 14 days after in vitro fertilization and must be discarded after this period. |
| 5.1.2 | | In vivo genetic manipulation of a human fetus is prohibited. |
| 5.1.3 | | Genetic manipulation to produce a transgenic human is prohibited. |
| 5.1.4 | | Genetic manipulation for the purpose of human enhancement and eugenics is prohibited. |
| 5.1.5 | | Before conducting the clinical trial of genetic manipulation, researchers should take into account safety considerations that include risk factors for patients, the community, and environment. Documentations for the safety of the study (such as studies on toxicity and tumorigenicity, exclusion of the possibility of gene transfer into the germline, and the absence of production of proliferative viruses in cases of using viral vectors) should be attached to the proposal of the clinical trial. |
| 5.1.6 | | Clinical trials of genetic manipulation in children (less than 18 years of age) are prohibited. This type of trial is allowed only in children who have life-threatening diseases with no alternative treatments. |
| 5.1.7 | | Genetic information obtained in the process of genetic manipulation of human cells should be considered confidential and all the principles of confidentiality should be observed by the researchers. |
| **5.2** | | Ethical considerations on genetic manipulation of animal embryos, fetuses, and cells |
| 5.2.1 | | Genetic manipulation of animal embryos, fetuses, and cells with the aim to create transgenic animals is allowed only when the goal is to treat diseases of humans or animals, and should not be carried out solely for the production of a new animal species. |
| 5.2.2 | | Transgenic animals should be kept under controlled conditions and necessary precautions should be taken to prevent their release into the environment. |
| 5.2.3 | | Genetic manipulation that causes suffering for animals or disrupting their normal life process by changing their traits and characteristics should be avoided. |
| Chapter 6 | | **Ethical guideline for research on tissue engineering in regenerative medicine** |
| 6.1 | | Clinical trials of tissue engineered products should be performed by taking into consideration the degree of interaction with the body, frequency and duration of the application, and transplantation conditions in accordance with the regulations approved by the Iran Food and Drug Administration. |
| 6.2 | | Tissue engineering clinical trials should be conducted under the supervision of a medical professional group. This group should be aware of the characteristics of the engineered components and product. |
| 6.3 | | Biomaterial design should be in such way that there is no danger to the researchers during the production and evaluation processes. |
| 6.4 | | Biohazardous materials that have been used in the production of biomaterials should be disposed of or recycled according to all safety guidlines approved by the Iran Food and Drug Administration. |
| 6.5 | | Research for changing a tissue for a purpose beyond what is necessary for health protection and tissue repair or replacement is prohibited. |
| 6.6 | | Production, storage, and application of cells for tissue engineering research and clinical trials should be carried out in compliance with the ethical considerations mentioned in other chapters of this guideline (ethical guidelines for research on stem cells, somatic cells, and clinical trials). |
| 6.7 | | Preparation and use of human tissues for tissue engineering applications must be carried out based on the ethical considerations mentioned in other chapters of the Ethical Guidelines for Research on Stem Cells and Regenerative Medicine and in compliance with the Ethical Guideline for Research on Human Organ and Tissue, approved by the Iranian Ministry of Health and Medical Education. |
| 6.8 | | Extraction of animal tissue and the use of animals as research models must be carried out in compliance with ethical considerations related to animals mentioned in the chapter of Ethical guideline for pre-clinical studies in regenerative medicine and in compliance with the Guideline for the Care and Use of Laboratory Animals, approved by the Iranian Ministry of Health and Medical Education. |
| Chapter 7 | | **Ethical guideline for pre-clinical studies in regenerative medicine** |
| **7.1** | | Choosing a suitable animal model |
| 7.1.1 | | If laboratory animal alternatives such as "organoid-derived pluripotent stem cells" are available, the use of animal models in research is not permitted. |
| 7.1.2 | | Pre-clinical studies should be conducted in appropriate animal models to produce valid information needed to design the clinical trials. Animal species selected for evaluation of biological activity and safety of regenerative medicine products must demonstrate the biological responses similar that expected in humans .. The rationale behind the pre-clinical study design and the criteria used to select a particular animal model should be justified scientifically and ethically. |
| 7.1.3 | | Animal species can be used if they are exclussivly bred to be used in the research. Wildlife and stray animals will endur tremendous stress and suffering after captivity, which is not acceptable ethically and with regards to scientific considerations. |
| 7.1.4 | | Non-standard animal species such as transgenic animals and those derived from genetic manipulations are acceptable for animal studies when the grounds and sufficient scientific justification for the use of this type of species is provided. |
| 7.1.5 | | Use of large animals in a study (e.g., primates, sheep, pig, goat, and horse) are allowed after providing sufficient evidence and scientific justification of the study purpose, which could not be achieved with a smaller animal species. |
| 7.1.6 | | Primate animals can be used only in studies carried out with an approach to the prevention, diagnosis or treatment of a disability or clinical conditions that are potentially hazardous to human life, and when there is sufficient scientific justification for the study and research purpose, which could not be achieved by using other animal species. |
| 7.1.7 | | Research on endangered animal species protected by the Department of Environment, Islamic Republic of Iran is prohibited. |
| 7.1.8 | | Sick animals that have become patient spotaneously and the disease is not scientifically induced, should not be used in the study. Such animals should undergo veterinary examinations as patients and be treated appropriately according to their conditions, or the appropriate decision should be made about the illness in the research project in accordance with the principles of working with animals. |
| 7.1.9 | | Pregnant or lactating animals should not be used in studies unless the Research Ethics Committee is certain that there is a sufficient scientific justification to conduct the study and this project is of utmost importance, such that failure to perform the study will lead to a major disruption in human or animal health. |
| **7.2** | | Animal interventions |
| 7.2.1 | | Surgery or dissection of animals or other interventions that inflict similar surgical pain on live non-anesthetized animals or those that do not undergo full analgesia is strictly prohibited. |
| 7.2.2 | | Animal interventions, especially those cause any pain or distress to the animal, should be done in the shortest possible time. |
| 7.2.3 | | While performing surgery and other invasive interventions on laboratory animals, the least invasive methods must be used, the minimal tissue manipulation and tissue damage should occur, and the intervention must be completed in the shortest possible time. |
| 7.2.4 | | All animals used in interventions, whether those kept individually or in groups, should be marked, if needed, using methods that induce the least amount of pain. |
| 7.2.5 | | Any anesthetic or analgesic drug use is considered a stress for the animal. Therefore, its frequency must be reduced as much as is practicable. |
| 7.2.6 | | Researchers need to estimate the amount of pain and distress of interventions in the animals before starting the exprimentand should treat them with using appropriate methods. Regular assessment and investigation must be taken into consideration to discover evidence of pain or distress in animals during and after the projects. |
| 7.2.7 | | No animal must be left alone after surgery or during anesthesia, and favorable conditions should be provided to help the animal return from anesthesia according to technical principles presented in valid and up-to-date literature such that no harm is imposed on the animals. |
| **7.3** | | Animal housing and place of studies |
| 7.3.1 | | Animal studies should not be conducted at the place for breeding or housing of the animals unless these studies are based on justified reasons approved by the Research Ethics Committee. In this case, it would be impossible to carry out the research projects outside the places for breeding or housing the animals. |
| 7.3.2 | | The place for animal studies must be designed and built based on the principles of good laboratory practice (GLP) approved by the Iran Food and Drug Administration or other international organizations. If any damage occurs to laboratory animals due to a lack of the necessary facilities in place for animal interventions, regardless of legal liability aspects, it is also considered an ethical violation. |
| 7.3.3 | | For each animal study, the study in compliance with the principles of GLP must be noted in the research proposal. If it is not possible to comply with the foregoing principles, the researchers must explain the reason so that the Research Ethics Committee can decide about the feasibility of the project. |
| 7.3.4 | | Laboratory animal care centers should provide the grounds for sharing organs and tissues of dead animals after the end of the study in order to reduce the number of animals killed. |
| 7.3.5 | | The procedure to keep animals, especially animals with more developed nervous system (including primates), should be such that the ground for psychological and behavioral comfort of the animals is ensured with environmental enrichment (simulating the natural living environment or even providing tools for the animal to play with). Also, abnormal bothering noises should be eliminated from the environment. |
| 7.3.6 | | Animal care practices (individual or group) and factors that include the needs of animal species, behavioral characteristics of each animal, requirements of research, animal health status, existing facilities on-site for maintenance and other factors that affect animal welfare should be taken into consideration. |
| 7.3.7 | | If the animals should be kept in individual cages, the time to separate the animal from its group should be reduced to the minimum time possible. For most species of animals, if an animal is kept alone, it must be able at least to see its species and feel their presence in its vicinity, either by senses of hearing or smell. Overall, dependence between animals of the same species should be taken into consideration. |
| 7.3.8 | | If the animals should be kept together as a group, it is necessary to prevent stress and aggressive behaviors by taking into consideration appropriate measures to eliminate these behaviors. The number of animals at the maintenance site must be determined based on regulations and standards presented in valid scientific resources and environmental and social conditions for each animal species should be considered. |
| 7.3.9 | | Research projects that require animals to be maintained under abnormal environmental conditions should be approved by the Research Ethics Committee prior to the beginning of the study. Adequate time for the animals to gradually adapt to these conditions must be considered. |
| 7.3.10 | | The animals should have ongoing access to safe drinking water and adequate food. |
| 7.3.11 | | Unnecessary animal confinement for extended periods of time should be avoided. |
| **7.4** | | Veterinary and care personnels |
| 7.4.1 | | All individuals involved in working with animals should be adequately trained, qualified, and undergo continuous training. |
| 7.4.2 | | Items such as availability, low cost, old age, or unusable for some animals cannot justify the fact that inexperienced individuals should work with them. |
| 7.4.3 | | The staff responsible for keeping the animals must have a moral obligation and treat the animals with tolerance when working with them, and respond appropriately in case of defensive and aggressive behaviors by the animals. They should restraint the animals correctly, and be aware of the importance of the research project and their role in animal health protection. |
| **7.5** | | The procedure to end working with animals |
| 7.5.1 | | Criteria and methods of ending the work with animals must be approved by the Research Ethics Committee. |
| 7.5.2 | | No animal should be abandoned after the end of the study and the final decision about ending the procedure must be made in accordance with the Guideline of Care and Use of Laboratory Animals (approved by the Iranian Ministry of Health and Medical Education). |
| 7.5.3 | | The end of working with an animal does not necessarily refer to killing the animal. Unreasonable killing of animals should be avoided as much as possible. However, keeping them alive should not cause unavoidable pain and suffering. |
| 7.5.4 | | The end of working with an animal that result in abandonment and spontaneous death of the animal is prohibited. |
| 7.5.5 | | If the research process leads to any lasting harm or pain, suffering, unforeseen and unnecessary distress in the animal, it should be stopped quickly. In this regard, prior to the research, criteria for the end of working on an animal must be specified and provide guidance for when the animal situation worsens during research and cannot be relieved or the animal situation cannot lead to scientifically valid results. |
| 7.5.6 | | Euthanasia of all kinds of animals after the completion of the study, at any age (embryo, fetus, larvae, baby, and adult) is a highly specialized intervention and must be carried out correctly so that it is associated with an uneventful, painless death with no suffering for the animals. |
| 7.5.7 | | The euthanasia method must be exactly selected based on technical criteria and principles presented in up-to-date scientific resources and must be approved by the Research Ethics Committee. |
| 7.5.8 | | The euthanasia location should be away from other animals so that they cannot hear any sounds, smell blood, and see the animals undergoing euthanasia (directly or indirectly). |
| 7.5.9 | | At the end of euthanasia, a qualified person is required to ensure the death according to the relevant standards of animal species. |
| 7.5.10 | | Animals’ carcasses and body parts are classified as Biohazardous waste and must be discarded in accordance with regulations that pertain to the administration of medical wastes by the Department of Environment, Islamic Republic of Iran. |
| Chapter 8 | | **Ethical guideline for clinical trials on stem cells and regenerative medicine** |
| **8.1** | | Harmlessness of clinical trials and the individual's health status |
| 8.1.1 | | Stem cells and regenerative medicine clinical trials can be carried out only when there is no effective treatment for the disease at the time of research or it is expected that the intervention will produce better results and fewer complications compared to the other existing therapies based on pre-clinical data and available scientific evidence. In cases where there is effective treatment, risks of interventions related to stem cells and regenerative medicine must be at a minimum and justifiable level, and their use leads to potential advantages such as fewer complications, shorter recovery time, increased quality of life, and reduced costs over the long-term. |
| 8.1.2 | | If the disease in question is severely debilitating or life-threatening, possible risks of clinical trials of stem cells and regenerative medicine in patients may be justified. However, maximum effort must have been made to minimize the risks and possible side effects. |
| 8.1.3 | | Clinical trials on vulnerable groups (such as children, pregnant women, mentally retarded persons, prisoners) can be done only when these trials bring about a direct benefit for the group in question. These trials must comply with the Ethical Guideline for Research on Vulnerable groups (approved by the Iranian Ministry of Health and Medical Education). |
| 8.1.4 | | Since the transplanted cell is a living organism and may remain in the body for a long time, accurate and continuous monitoring of patients with greater sensitivity and precision than other clinical trials is required after transplantation. The duration and process of monitoring should be specified in the clinical trial protocol. |
| 8.1.5 | | Efficacy and, especially, the safety of cell-based products should be examined and verified in appropriate in vitro and animal models before starting the clinical trial. |
| 8.1.6 | | The results of animal studies (even when human cells are transplanted into animals) are not necessarily indicative of cell behavior in human. Safety and efficacy studies in animals may not be able to correctly predict the response in humans. For this reason, it is recommended to take all necessary measures for a detailed review of adverse effects in humans and confront to them. |
| 8.1.7 | | Collection, analysis, storage, or reuse of residual tissues obtained during diagnostic,therapeutic, and surgical procedures when the identity of the owner is known or can be traced and detected is possible only after obtaining informed consent from that patient or his/her legal representative. In cases where obtaining consent is impossible or may violate the validity of the research, only after Ethics Committee approval and respect to confidentiallity of the identity of owners, they can be used without obtaining informed consent. |
| 8.1.8 | | Clinical trial participants should not be deprived of conventional treatments merely for research purposes. |
| 8.1.9 | | As long as cell therapies are in the trial phase, the participants should not incur any of the costs of the research. If the Iran Food and Drug Administration conditionally approve the marketing of a cell-based product before the end of the trial phases, payment by the patient will be allowed only with the approval of the National Biomedical Research Ethics Committee. |
| 8.1.10 | | After the end of the clinical trials, if the use of cells and their products have not been confirmed by official authorities, their application in patients will not be permitted. |
| 8.1.11 | | Patients should not be given false hope in order to increase participation in clinical trials. |
| 8.1.12 | | Research investigators or research sponsor institutions should compensate for the damages imposed on the participants resulting from research. These include physical, financial or spiritual damages in accordance with the law and by opinion polling of competent authorities. |
| 8.1.13 | | The existence of any financial and non-financial relationship (excluding the clinical trial contract) with funders should be clearly stated in the project proposal by the principal invstigator or any of his/her colleagues. If necessary, the documentation should be attached to the project proposal. |
| **8.2** | | Considerations on donation of biological samples in clinical trial |
| 8.2.1 | | The cell and tissue donation process must cause a "minimal acceptable risk" to the donor. |
| 8.2.2 | | It is necessary to make optimum use of donated human organs and tissues, and prevent their loss such that a need does not exist to obtain more tissue samples. |
| 8.2.3 | | Demands and interests of the biological sample donor take priority over demands and interests of biological sample recipient, therefore, the donor should provide written informed consent. |
| 8.2.4 | | Obtained cells or tissues are dedicated to the donor and during any time of the study, the donor can withdraw from using his /her donated sample(s) and in this condition, it is not allowed to continue research on the donated biological sample. However, the statement of the withdrawal after the beginning of sample processing will impose additional costs on the research; therefore, the donor must be informed that he/she can withdrawal from the research just before the start of the sample processing. |
| 8.2.5 | | If the donor withdraws his/her consent to use the donated biologocal sample(s), the donor should not be charged or deprived of routine health care services. |
| 8.2.6 | | The biological sampling of aborted fetus can be performed after obtaining the informed consent of both parents. However, taking the samples of other pregnancy products (e.g., umbilical cord, cord blood, placenta, and amniotic fluid) can be done after obtaining the inform consent of the mother or her legal representative. |
| 8.2.7 | | Cadaveric tissue sampling should be in accordance with national regulations and in compliance with the Ethical Guideline for Research on Human Organ and Tissue (approved by the Iranian Ministry of Health and Medical Education). |
| 8.2.8 | | Any financial relationship between the donor and recipient in cell transplantation and its products is not allowed. |
| 8.2.9 | | Any special financial incentive and concession to create incentives for the donation of cells and tissues is prohibited. The researcher can only offset the costs imposed on the participant for participation in the research (e.g., travel expenses) in the form of non-cash gifts. |
| **8.3** | | Designing a clinical trial |
| 8.3.1 | | A randomized double-blind, placebo-controlled trial (usually after phase 1) takes priority when designing a clinical trial. This means that if it is scientifically and practically feasible to design a double-blind randomized controlled trial, other designs will not be accepted. |
| 8.3.2 | | The next priorities for designing a clinical trial (usually after phase 1), respectively, include randomized single-blind, placebo-controlled trial, controlled clinical trial (without blinding), and clinical trial without control (without blinding). |
| **8.4** | | Primary and secondary outcomes of clinical trials |
| 8.4.1 | | Primary and secondary clinical outcomes in terms of safety and efficacy of clinical trials on stem cells and regenerative medicine should be presented separately and clearly. Recording and reporting levels of adverse effects should be based on national and international guidelines. |
| **8.5** | | Ensuring the safety of clinical interventions |
| 8.5.1 | | Pre-clinical evaluations: The safety and potential risks of cells should be evaluated to reduce related complications and risks before any cell-based clinical trials. This assessment includes evaluation of resources and raw materials used, production steps, qualification steps, and measures related to the safety of the intermediate and final product. Ethical considerations listed in the chapter: "Ethical guideline for pre-clinical studies in regenerative medicine" is essential for pre-clinical evaluation. |
| 8.5.2 | | Clinical evaluation: In order to carry out clinical trials for stem cells and regenerative medicine products, it is necessary to perform clinical assessments based on national and international standards, and in compliance with national ethical guidelines in research (approved by the Iranian Ministry of Health and Medical Education). Special considerations for clinical assessments are as **follows**: |
| 8.5.2.1 | | Biological characteristics of cell therapy and regenerative medicine approaches used in clinical trials should be determined. |
| 8.5.2.2 | | Cell manufacturing process (consistent with manufacturing standards including Good Manufacturing Practice, etc.) and control of all resources used in the production of cellular products, including raw tissues, viral and non-viral vectors, enzymes, culture media, growth factors, and other necessary additives as well as sufficient current information that pertains to cellular clinical intervention risks such as potency of cell proliferation, results on the assessment of the likelihood of tumorigenesis in animal models, and risks associated with vectors viral should be determined. |
| 8.5.2.3 | | Data from pre-clinical studies, animal models, and other suitable models should be considered to evaluate safety and effectiveness prior to conducting the clinical trials. |
| 8.5.2.4 | | Researchers should offer the participants acceptable evidence concerning the benefits and possible complications of participating in the clinical trial. This information should be clearly transferred to the participant during the process of obtaining informed consent. Explanations should be also given to participants about the benefits and complications of new treatment with the cell therapy and regenerative medicine approaches (given that patients may have access to alternative and conventional therapies) such that participants, to the extent possible, avoid potential misunderstandings about the effects of this approach. |
| 8.5.2.5 | | It is prohibited to carry out sham interventions in clinical trials of stem cells and regenerative medicine except in cases where the risks and possible complications are accurately evaluated and approved by the Research Ethics Committee. |
| 8.5.2.6 | | Researchers should have a systematic program for the treatment and management of possible complications such as toxicity, tumorigenesis, and other complications of the clinical intervention. |
| 8.5.2.7 | | It is necessary to design controlled clinical trials, especially for phase-III clinical trials, in order to perform a detailed comparative study on the effects of cellular interventions and its products. |
| 8.5.2.8 | | Measurable variables and parameters must be defined clearly. |
| 8.5.2.9 | | It is necessary to ensure the availability of supportive resources to compensate for any adverse effects resulting from interventions during the trial. |
| **8.6** | | First-in-human clinical trial requirements |
| 8.6.1 | | The participants should be informed that this clinical trial of stem cells and regenerative medicine has never been conducted in human. |
| 8.6.2 | | Sufficient information should be offered to participants about the profit and risk, and possible complications and limitations of this clinical trial. |
| 8.6.3 | | Participants of first-in-human clinical trials of stem cells and regenerative medicine should have end stage of their diseases. However, they should be so incapable of taking part in a clinical trial brings about irreparable threat to them. |
| 8.6.4 | | Attempts should be made to ensure participants’ understanding (commensurate with their extent of knowledge) about the complex process of the trial as well as the authenticity of the process of obtaining informed free consent. |
| **8.7** | | Publication of clinical trial results |
| 8.7.1 | | Publication of positive and negative results (efficiency or inefficiency) and all complications should be performed as appropriate to enable access and use by other researchers and institutions active in this area. The results must be published in compliance with Ethical Guideline for the Research Publication (approved by the Iranian Ministry of Health and Medical Education). |
| Chapter 9 | | **Ethical guideline for stem cells and regenerative medicine bio-banks** |
| **9.1** | | Collection, management, and access to biological samples |
| 9.1.1 | | Biological samples must be kept safely and should be labeled or coded. They should be maintained such that they are not damaged or lost, and unauthorized persons do not have access to these samples. |
| 9.1.2 | | Biological samples should be stored and used for research considering ethical and legal principals. They should not be collected and used for other than defined purposes. |
| 9.1.3 | | Biological samples belong to donors; however, bio-bank officials are allowed to possess and manage samples based on laws and regulations according to an agreement reached with the donors. |
| 9.1.4 | | Private bio-banks can charge costs from donors for maintenance and protection purposes, and are responsible for the maintenance of these biological samples. |
| **9.2** | | Informed consent |
| 9.2.1 | | Informed consent must be obtained before donation of biological samples from the donor or his/her legal representative. This consent must be informed freely, and obtained receipt of adequate information about the methods and purposes of the research or treatment, and the potential risks and benefits. Donors should be opinion polled about how to provide possible results of their research. |
| 9.2.2 | | In case of obtaining biological samples from people who lack decision-making capacity, their legal representatives can use discretion to consent on behalf of those individuals to donate their biological samples. |
| 9.2.3 | | Biological samples from children (those who have not reached the legal age) can only be obtained with the consent of the child’s parents or their legal guardian. Oral consent for children 7 to 14 years of age, and written consent for children ages 14 to 18 years is required. |
| 9.2.4 | | In case of obtaining biological samples from aborted fetuses, the consent of both parents is required. With regards to donating other products of conception (such as umbilical cord, cord blood, placenta, and amniotic fluid), maternal consent is sufficient. |
| 9.2.5 | | The donor can withdraw his/her consent in writing and call for the destruction of the donated sample before the start of sample processing. If they announce the intent to withdraw from the study after processing of their biological samples, additional costs will be imposed on the bio-banks. Thus, an agreement must be made with the donor in this regard when obtaining consent. Withdrawal of consent is possible only in cases of identified samples. |
| 9.2.6 | | If the bio-bank modifies or expands the scope of its activities, it must obtain consent from the donors again.  *Note 1*: In case of population-based studies where obtaining consent is impossible, the National Biomedical Research Ethics Committee is allowed to make decisions about the need to obtain consent.  *Note 2*: In case of using anonymous data, measures should be taken in accordance with the provisions of the initial consent. |
| 9.2.7 | | In case of death of the biological sample donor, the anonymization process must be carried out on the biological sample. |
| **9.3** | | Data protection |
| 9.3.1 | | In each bio-bank, individuals who are allowed to access the samples should be authorized. |
| 9.3.2 | | Bio-bank staff that has access to the samples and biological data must respect privacy policies and avoid data transfer without legal authorization. |
| 9.3.3 | | The information technology section of each bio-bank is required to apply technical standards for protection of data and biological samples. |
| Chapter 10 | | **Ethical guideline for privacy and confidentiality** |
| 10.1 | | The research ethics committees should investigate the confidentiality of the protocol, while reviewing research proposals on stem cells and regenerative medicine to safeguard the privacy and confidentiality of participants. |
| 10.2 | | The research proposals should be designed in such a way that the need to collect the identifiable information of participants is minimized. The protocol of access to information should be defined based on the "need to know" and "minimum necessary" standards. |
| 10.3 | | In cases in which collecting identifiable information of research participants is necessary, the researcher must predict and present a clear protocol to protect the confidentiality of the identifiable data and data storage security. Research ethics committees are required to ensure the existence and implementation of this protocol. |
| 10.4 | | The researcher has a responsibility to protect donors and participants' information. At the same time, all members of the research team are obligated to observe the principles of confidentiality and privacy. |
| 10.5 | | In cases where donated biological samples are screened in terms of infectious diseases or genetic disorders, the researcher must reach an agreement with the donor while obtaining informed consent as to whether he/she would like to be notified of the results and how this notification must be performed. |
| 10.6 | | Electronic data, including identifiable data of biological samples, must be protected with the highest security standards such as coding, storage of data in a secure room, lack of an internet connection to avoid data hacking, minimum access, and determining the level of access. |
| 10.7 | | Researchers should anticipate the necessary facilities for maintaining the confidentiality of information. |
| 10.8 | | It is necessary to protect confidential information when using donated tissues or cells from the body of a deceased donor and surplus or disposable tissues obtained from diagnostic and therapeutic interventions. |
| **Chapter 11** | | Ethical guideline for obtaining informed consent |
| **11.1** | | Informed consent to donate biological samples |
| 11.1.1 | | It is necessary to obtain transparent and updated informed consent for the use of all biological samples. This consent must be obtained before collecting biological samples for both the present research and possible future research. |
| 11.1.2 | | Transparent and up-to-date consent means that donors should give their consent regarding how their samples should be used in research processes before providing these samples. |
| 11.1.3 | | When biological samples are received from a bio-bank, the researcher is not required to re-obtain consent from the donor. However, bio-banks are responsible to obtain appropriate informed consent from donors while storing their samples and transfer a copy of informed consent documents to researchers when requested. |
| 11.1.4 | | It is necessary to obtain informed consent for research use of donated biological samples obtained from clinical diagnostic and therapeutic interventions. The process should be completely separated from the process of obtaining the consent for diagnostic or therapeutic interventions. In order to guarantee this goal and also ensure free choice by donors, the responsible person for these two processes should not be the same. |
| 11.1.5 | | Objection to the use of biological samples obtained for therapeutic interventions in research should not affect diagnosis and treatment of the patient under any circumstances. Patient treatment and diagnostic protocols should not change in this situation or the patient should not be deprived of necessary standards of care. |
| 11.1.6 | | It is necessary to obtain informed consent for research use of biological sample residues obtained from clinical diagnostic and therapeutic interventions, which is supposed to be discarded. |
| 11.1.7 | | If there is a possibility of access to important information about the health of the donor during the research process, the researcher should specify his/her protocol on sharing the results prior to the research. |
| 11.1.8 | | The consent to donate an embryo or fetus should be taken from both parents. If there is third party (gamete donor), consent to donate the embryo or fetus must also be obtained from him/her. This consent must be obtained "after the decision to withdraw the use of the embryos for infertility treatment" or "after the abortion". |
| 11.1.9 | | While obtaining the consent to donate aborted fetuses, the parents should be asked about their tendency "to take back the fetus after obtaining the sample". According to the consent of the parents and their request, if possible, the fetus should be returned to them after the end of the study or to be buried by the research team in conformity with the legal and religious rituals (fetuses with developed organs should be buried). |
| 11.1.10 | | Maternal consent to donate the products of conception (such as umbilical cord, cord blood, placenta and amniotic fluid) is necessary. |
| 11.1.11 | | If the donor lacks the capacity to consent or could not consent voluntarily (such as children, psychiatric patients, mentally disabled persons, dementia patients, prisoners), it is forbidden to obtain biological samples unless there would be a health benefit to the donor. In case of prisoners, the consent should be obtained only from the prisoner himself/herself. |
| 11.1.12 | | Consent to donate biological samples from children (those who have not reached the legal age) must be obtained from the child's parents or their legal guardian. In the case of children between the ages of 7 and 14 years, the assent of the child is acceptable; in the age group 14 to 18 years, child's written consent is also required. |
| 11.1.13 | | If the donor is mentally disabled but has some degree of decision-making capacity, the consent should be obtained from the donor. In such circumstances, an expert (such as a psychologist) must assess the donor’s decision-making capacity. |
| 11.1.14 | | If a deceased or brain-dead person expressed his/her wishes to donate tissues and organs for research during his/her lifetime, then their first-degree relatives or legal guardians can provide consent for research use of the biological samples. If the deceased or brain-dead individuals did not express their consent or disagreed with this issue, then consent cannot be given by the first-degree relatives or legal guardians to donate the biological samples. |
| 11.1.15 | | Any use of a brain-dead patient’s organs should be made after donation of vital tissues for treatment purposes unless the patient indicated, during his/her lifetime, that his/her tissues should be used for research. The decision on cessation of cardiac and respiratory support in patients who are brain dead should not be made for research purposes of the body's tissues retrieval. Researchers should not be the decision makers in this regard. Generally, brain death approvalprotocols as well as legal issues related to brain death should be observed for these donors. |
| 11.1.16 | | For donation of biological samples, the **following issues** should be mentioned in the informed consent form: |
| 11.1.16.1 | | The research objectives, methods of sampling, number and type of donated samples, intended use of samples, and risks of donation should be explained. |
| 11.1.16.2 | | Disposal or storage of the samples after the completion of the study and, if possible, the duration of storage should be specified. |
| 11.1.16.3 | | Determining whether there is a possibility for long-term storage of donated samples to be used for future research. |
| 11.1.16.4 | | How the remains of the donated samples will be disposed. |
| 11.1.16.5 | | Whether or not the donor will be contacted in the future for newer studies, obtaining more samples, or for additional information. |
| 11.1.16.6 | | Whether the donated samples will undergo genetic manipulation or not. |
| 11.1.16.7 | | Whether the donated sample will undergo a screening process in terms of infectious or genetic diseases or not. In case of genetic screening, sufficient explanation should be given to the donors about the type of tests that will be carried out on the sample, the procedure to reveal the results of screening, and how to maintain the confidentiality of the screening results. |
| 11.1.16.8 | | The manner of revealing the results of the biological samples, the confidentiality of the donor's personal information, and, if possible, the time frame for deleting the information should be provided. |
| 11.1.16.9 | | If the donor may or may not have medical benefits from research on their donated samples. |
| 11.1.16.10 | | Donations may lead to the production of a product that yields financial gains. The research team or the organizations that support the research have the right to benefit from the financial gains and other intellectual properties of the research. |
| 11.1.16.11 | | Consent to donate samples for research or rejecting the donation does not affect his/her health services. |
| 11.1.16.12 | | Donors of biological samples can withdraw their consent at any time of the study. In these circumstances, researchers will not be allowed to continue research on the donated samples and the samples should be discarded. If possible, the samples should be returned to the donors in accordance with the agreement. However, since withdrawal after the beginning of sample processing will impose additional costs on the research, it must be done before the start of any sample processing. |
| 11.1.16.13 | | If each parent withdraws consent from donating the aborted fetus at any time during the study, the use of the fetus in research must be stopped and the fetus should be returned to his/her parents upon their request or buried according to legal and religious rituals. |
| 11.1.16.14 | | The donated embryos will never be used for fertility purposes for another person. |
| **11.2** | | Informed consent to participate in research |
| 11.2.1 | | Obtaining informed consent is necessary in all stem cell and regenerative medicine researches. If the participants do not have the capacity to consent, their surrogate decision maker should provide consent. |
| 11.2.2 | | Understanding and decision-making capacity of the participants, especially those with the diseases or conditions that affect their cognitive capacities, should be evaluated before obtaining consent. |
| 11.2.3 | | When doing research on people who lack capacity to make decisions, even in case of the consent of the surrogate decision-maker, complications caused by participating in the research should not be more than that of "minimal acceptable risk". |
| 11.2.4 | | If any changes occur in the research protocol during the study or new findings suggest new benefits and risks due to intervention, the participants should be informed and a new consent must be obtained. |
| 11.2.5 | | If during the research process, there is the possibility of access to important information about the participant’s health status, the researcher must determine an agreed approach to inform the participant. |
| 11.2.6 | | Unlike other pharmaceutical and clinical procedures such as surgery, both stem cells and regenerative medicine-based interventions can have lasting effects on the structure and function of the human body that may persist throughout life. Therefore, it is necessary to clearly explain the possible irreversible consequences of these interventions to participants, especially in the case of cell transplants. |
| 11.2.7 | | Those involved in obtaining informed consent and processing of tissues and cells should be independent of the treatment team. |
| 11.2.8 | | To honor the values and beliefs of people, participants should be informed of the origin of biological materials that will be used in the study. |
| 11.2.9 | | In order to achieve a better understanding of the processes that occurs in the body, it is better to obtain participants’ consent to allow researchers to perform whole or restricted autopsies in case of their death for any cause and at any time of the research, so that researchers can investigate the consequences of intervention in the long term. It is obvious that requesting for an autopsy largely depends on individuals' beliefs and cultural characteristics. |
| 11.2.10 | | While obtaining informed consent, especially in the early phases of clinical trials, it should be noted that participants may have an optimistic view regarding the potential benefits and possible therapeutic effects of the intervention. Severe clinical conditions make these individuals prone to misunderstanding about the benefits of participating in the study. In such circumstances, it is the duty of the researcher to ensure participants' understanding of the conditions and their informed consent to participate in the research by emphasizing the experimental nature of the intervention. The **following precautions** will be useful in this regard: |
| 11.2.10.1 | | The process of obtaining informed consent must be carried out by someone other than the research team. |
| 11.2.10.2 | | Participants should have sufficient time to make their decisions. |
| 11.2.10.3 | | Participants' views about the therapeutic benefits of the intervention must be evaluated before obtaining informed consent. |
| 11.2.10.4 | | It must be explained to the participants that it is rarely possible to access definitive treatment benefits at this stage of the research. |
| 11.2.10.5 | | When describing the research protocol, words with treatment connotations should not be used as much as possible. |
| 11.2.10.6 | | Separate written educational materials on the subject should be made available to the participants. |
| 11.2.11 | | Informed consent form to participate in the study should include at least the **following issues**: |
| 11.2.11.1 | | Profile of the researcher, the research organization, and sponsor organizations (introducing the physician or medical group who are directly engaged in the clinical trial). |
| 11.2.11.2 | | The aim of the study and its phases should be expressed in plain and understandable language. |
| 11.2.11.3 | | Predicting the effectiveness of cell therapy and regenerative medicine approaches in comparison with other therapeutic alternatives based on available scientific information. |
| 11.2.11.4 | | Type, number, and characteristics of cells or cell-based products which received. |
| 11.2.11.5 | | Benefits and risks of participating in the study from the beginning to the end of the study and the intervention protocol in case of any complications. |
| 11.2.11.6 | | Informing about laboratory tests (including screening tests for infectious diseases and genetic disorders) and necessary physical exams to check participants’ health status and their right to know the results. Of note, participants need to be informed already about tests and examinations. Necessary explanations about the possible limitations of diagnostic tests to detect diseases should be given. |
| 11.2.11.7 | | Type and amount of the participant’s involvement. |
| 11.2.11.8 | | The participant’s agreement for future calls to report possible outcomes or receive additional information. |
| 11.2.11.9 | | Voluntary participation in the research process and the right to withdraw from the research without affecting their diagnostic and therapeutic care. |
| 11.2.11.10 | | Emphasizing the participant’s confidentiality and privacy and the way for revealing the results to them. |
| 11.2.11.11 | | Participating in research will have no cost to the participants (e.g., free interventions). |
| 11.2.11.12 | | The compensation protocol for potential complications due to participation in the research. |
| 11.2.11.13 | | Contact number and address of the Principal Investigator. |
| 11.2.11.14 | | Contact number and address of the Research Ethics Committee (committees that approved the project or committees that are references for the research complaints). |
